# Proficiency based progression simulation training significantly reduces utility strikes; A prospective, randomized and blinded study

**DOI:** 10.1371/journal.pone.0231979

**Published:** 2020-05-12

**Authors:** Anthony G. Gallagher, Martin Hart, David Cleary, Craig Hamilton, Kevin McGlinchey, Patrick Kiely, Brendan P. Bunting

**Affiliations:** 1 Faculty of Life and Health Sciences, Ulster University, Magee Campus, Londonderry, Northern Ireland, United Kingdom; 2 Group Training and Development Manager, ReachActive Unit 4B Lough Sheever Corporate Park, Mullingar, Co. Westmeath, Ireland; 3 ReachActive, Mullingar, Co. Westmeath, Ireland; 4 ReachActive, Hertford, England, United Kingdom; 5 Flux Learning Ltd., Clonakilty, Co Cork, Ireland; 6 School of Psychology, Coleraine, Co. Londonderry, Northern Ireland, United Kingdom; Xiamen University, CHINA

## Abstract

**Objectives:**

We evaluated a simulation-based training curriculum with quantitatively defined performance benchmarks for utility workers location and excavation of utility services.

**Background:**

Damaging buried utilities is associated with considerable safety risks to workers and substantial cost to employers.

**Methods:**

In a prospective, randomized and blinded study we assessed the impact of Proficiency Based Progression (PBP) simulation training on the location and excavation of utility services work.

**Results:**

PBP simulation training reduced performance errors (33%, p = 0.006) in comparison a standard trained group. When implemented across all workers in the same division there was a 35–61% reduction in utility strikes (p = 0.028) and an estimated cost saving of £116,000 –£2,175,000 in the 12 months (47,000 work hours) studied.

**Conclusions:**

The magnitude of the training benefit of PBP simulation training in the utilities sector appears to be the same as it is in surgery, cardiology and procedure-based medicine.

**Application:**

Quality-assured utility worker simulation training significantly reduces utility damage and associated costs.

## Introduction

A feature in almost all construction projects is the need to carry out excavations or earthworks. Coupled with the fact that the vast majority of the UK’s utilities (gas, water, sewer, electric, telecoms) are located underground, these activities bring with them the principal risk of hitting and damaging this buried infrastructure Metje, Ahmad [1]. The potential danger and risks associated with work in this environment are not insignificant. See https://www.youtube.com/watch?v=bpX3VHKAKak for an illustration of the potential risks to utilities workers of an electrical strike. The highest rate of fatal electrical injury in 2016 occurred in the Utility industry i.e., 0.87/100,000 [2] and utility workers are at continuous risk of injury and life-changing burns [3]

A utility strike has been defined as occurring when any utility network infrastructure (i.e., electricity, gas, telecommunications and fresh or waste water) is hit and damaged during an excavation [2]. In a survey of its members The Utility Strike Avoidance Group (USAG) analysed over 2000 utility strikes in UK during 2014 from 24 participant groups. They concluded that the average strike cost estimates typically ranged between £2000 and £7,500 (mean = £3,600). Makana, Metje [2] in their analysis of sixteen more detailed case studies involving utility strikes suggest that the true cost of utility strikes might be significantly higher. They estimated that for every utility strike incurring a direct cost of £1,000, the true cost including indirect and social costs is in fact £29,000. This analysis would suggest that the true costs of the typical utility strike in the UK would be between £58,000–£217,500 [2]. In a subsequent report and based on further analysis Makana, Metje [4] re-estimated the average cost of a strike and concluded that it is ~£100,000 per strike when all associated costs are included.

**Fig 1:**
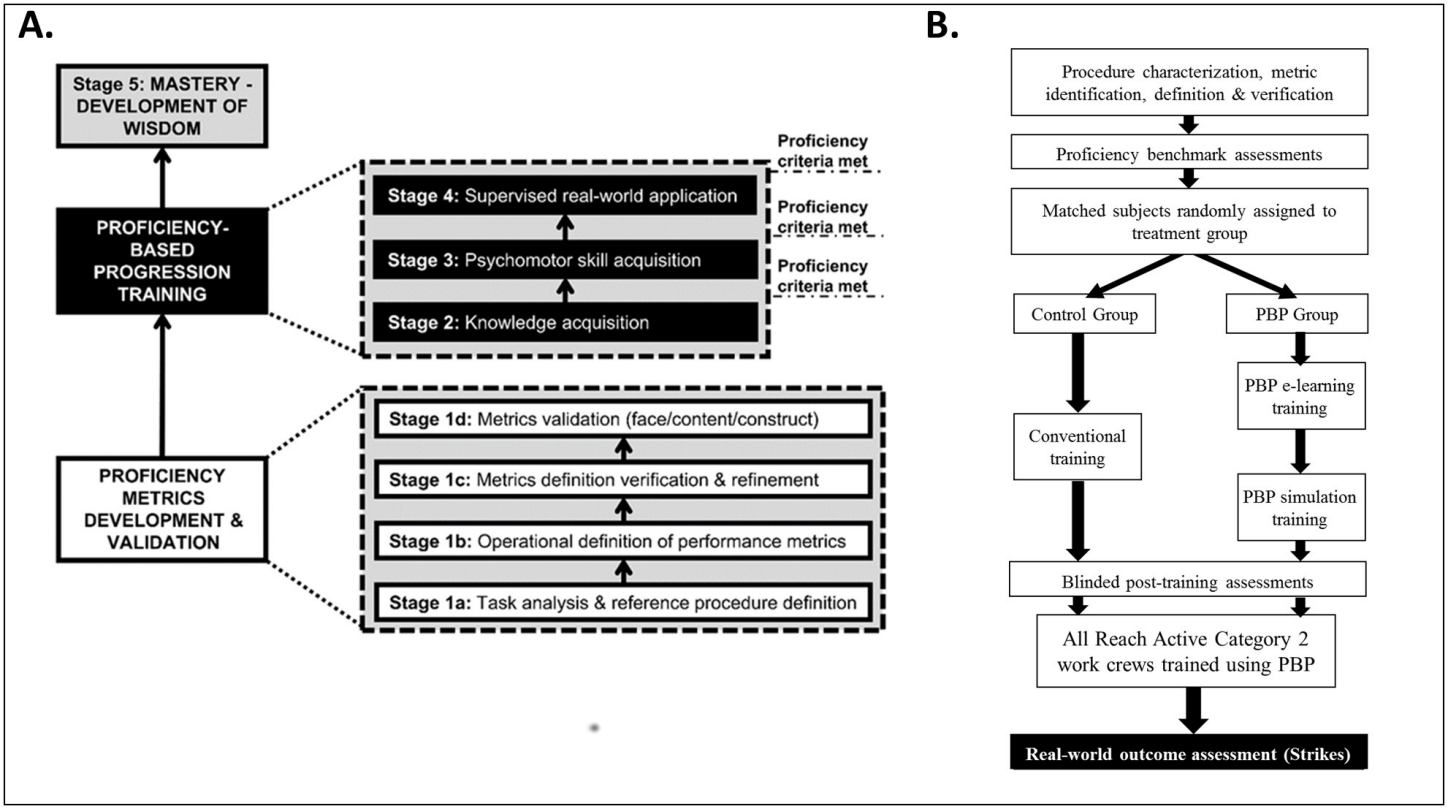
A. The Proficiency-Based Progression (PBP) training paradigm [11]. B. The outline of the prospective, randomised and blinded study.

Proficiency Based Progression (PBP) simulation training [5] is a method that has been applied for training skills in another high-risk environments, i.e., surgery and procedure based medicine. This approach to skill acquisition differs from conventional training, because the trainee must verify that they know what to do and can do it to quantitatively defined proficiency benchmarks which are objective, transparent and fair, before execution of their skills on the real world tasks. Furthermore, the performance metrics and proficiency benchmarks which underpin training are derived from practitioners who are very experienced and ‘proficient’ in the task or skills to be learned [6]. In prospective, randomised, blinded and multi-centre clinical trials PBP simulation training has been demonstrated to be an effective and efficient approach to education and training with operative performances that are >40% better than comparative training outcomes [7–10]. A PBP approach to training skills for working in a high risk environment has however never been used outside the field of clinical medicine. The method is outlined in Fig 1A. The metrics are first identified from a detailed task analysis from experienced practitioners who are ‘good’ at performing the procedure to be characterised (stages 1a-d). The metrics are then validated (i.e., face, content and construct validation) and once validated are used to build a curriculum (stages 2–4). Each stage of the curriculum has a performance/proficiency benchmark which trainees must demonstrate before progression to the next stage in their training. The quantitatively defined performance/proficiency benchmarks are derived from the performances of experienced practitioners who are actually ‘good’ at performing the same procedure. Benchmarks are quantitatively defined on the mean performance of the experienced and ‘good’ practitioners. Experienced practitioners scores whose performance is atypical to their peers (i.e., more than two standard deviations from the mean scores of their peers) are excluded from the proficiency benchmarking [12–14].

**Aims**: The aim of this study was to assess if the application of a PBP training curriculum for the location and excavation of underground utility services resulted in a significant reduction in the number of performance errors when compared to current training methodologies. If PBP training did positively impact on performance, the goal was then to roll out this approach to training and skill verification to an entire division within the organization and then evaluate its impact on utility strike rates.

## Materials and methods

### Procedure

The study was a prospective, randomized and blinded controlled trial as shown in the design outlined in Fig 1B. The control group received the current and conventional training and the PBP trained group went through the PBP training curriculum as described elsewhere [5, 11].

This research was approved by the Institutional Review Board Clinical Ethics Research Committee of the Cork University Teaching Hospitals (ECM 4 (h) 17/10/2017 and ECM 3 (ww) 7/11/2017). Informed consent was obtained from each participant.

A detailed procedure characterisation of ‘mark-up and digging’ was conducted (during 2017), which included the identification and operational definition of optimal and suboptimal performance (i.e., deviations from optimal performance or errors) and more serious deviations from optimal performance that potentially exposed the workers or their colleagues to safety risks. The PBP process also quantitatively defined proficiency benchmarks based on the objectively assessed performance of experts, rather than just minimum competency performance levels or an estimated performance pass mark. Two comprehensive procedural checklists were derived from a detailed task analysis supported with video recordings of task performance. The Hand Digging Procedural Checklist consisted of three phases and 38 behavioural steps. This covered: Site Documentation and Personal Protective Equipment (13 steps with 13 potential Critical Errors); Digging Trial Holes (10 steps, with 4 potential Errors and 6 potential Critical Errors); and Commence Handing Digging (15 steps with 5 potential Errors and 10 potential Critical Errors).

The Scanning and Mark-Up Procedural Checklist, consisted of three Phases and 45 behavioural steps. This covered: Site Documentation and Personal Protective Equipment (13 metrics, with 13 potential Critical Errors); Visual Inspection and Mark-up (27 metrics with 5 potential Errors and 22 potential Critical Errors); and Additional General Steps for Visual Inspection and Mark-up (5 steps with 2 potential Errors and 3 potential Critical Errors). The checklist guided the construction of the e-learning and simulation training curriculum and the assessments which quantitatively defined proficiency benchmarks by two very experienced RA staff recognised for their skill and expertise on these tasks. One of the experts worked in Ireland and one working in London and the South East of England who repeated the assessment for benchmarking twice.

#### E-learning modules

**Fig 2:**
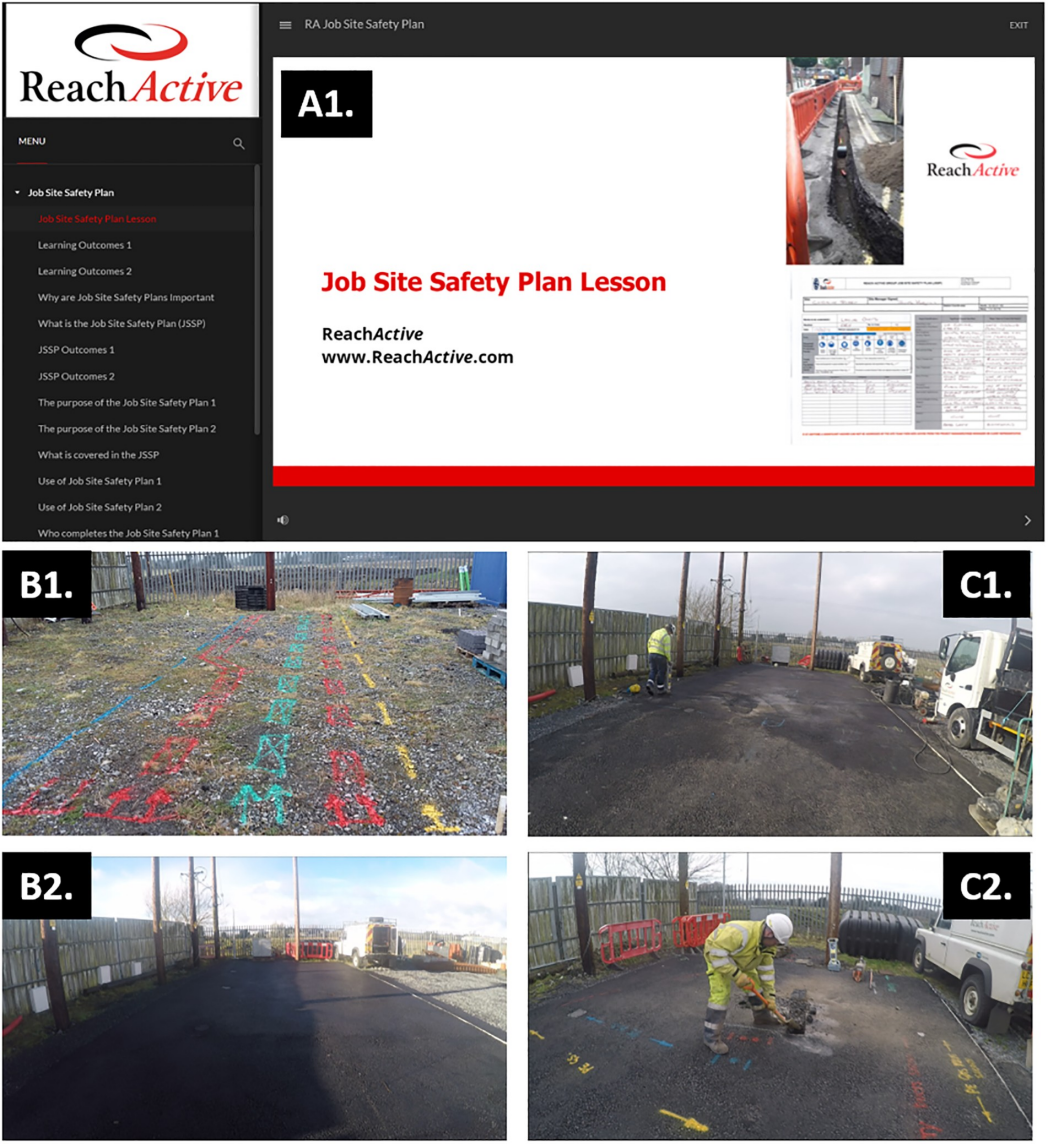
**A**. The graphical user interface of the ‘Job Site Safety Plan’, one of the modules for the e-learning component of the study. **B1**. The layout and configuration of the pipe and ducting utilities tasks in the simulation training area before being placed in the ground. **B2**. The final layout of simulation training area with the pipe and ducting utilities concealed beneath tarmac; **C1**. A PBP trainee working on the simulation for mark-up, location and identification of utilities in the ground with a Cable Avoidance Tool (CAT) tool. **C2**. The hand digging and excavation of pre-existing utility services that they have identified.

Online learning courses (Fig 2A1) were used to deliver the didactic and knowledge components of the PBP training. Flux Learning—the online learning provider—developed three courses 1) Job Site Safety Plans 2) Utility Plans 3) Visual Inspection using the commercial rapid authoring software and published to the SCORM 2004 standard. These courses delivered the content to the learners in a standardised format which included animated narrated walkthroughs of key task requirements. Subjects also had to complete a multiple choice question style assessment before proceeding to the next educational component. This enabled a proficiency benchmark to be established. The pass mark for each of the three courses was set at 90 percent. These courses commenced in February 2018 and were completed by 16 February 2018. Learning analytics were used to track each subject for time taken to complete, answers provided and time spent per question.

#### Procedural skills training

To address the procedural skills element of the training, an underground services training simulation area was constructed (shown in Fig 2B & 2C) which included all the services, power, gas, water, and telecommunications a utility operative might encounter. In addition, various other elements were added which would be important for the visual assessment process including underground chambers and valves as well as streetlamps, gas and meter boxes as well as overhead lines.

To address the procedural skills element of the training, an underground services training simulation area was constructed (shown in Fig 2B & 2C) which included all the services, power, gas, water, and telecommunications a utility operative might encounter. In addition, various other elements were added which would be important for the visual assessment process including underground chambers and valves as well as streetlamps, gas and meter boxes as well as overhead lines.

Simulation training for participants randomised to the PBP training group began only after they had successfully completed all three e-learning modules (beginning in February 2018, and all participants had successfully completed their simulation skills training by 26^th^ February).

### Subjects

Subjects recruited to participate in the study were drawn from ERA employees in the Civils Division who were working on the repair and maintenance of the ESB underground electrical distribution network in Dublin city and the urban area. Originally 17 participants were identified to participate in the study. These employees were briefed and their participation in the study was described and then their written consent to participate was obtained.

All employees in the study completed an online colour blindness test http://enchroma.com/test/. One employee was excluded because they were colour blind, but the results for number of other employees were inconclusive. All remaining employees were asked to identify the colours on a number of utility plans that would typically be used on a site excavation and were able to identify the all of the colours successfully.

In addition, another employee had to be excluded from the study when it was identified that they did not possess a Construction Skills Certification Scheme (CSCS) accreditation for the Location of Underground Services, which was a mandatory requirement for the use of a Cable Avoidance Tool (CAT) and Genny. A further employee left the company shortly after process characterisation phase and two more employees were assigned to other duties by the time of the study stage. This meant that there were 12 employees that were available to be randomly assigned to the Control or PBP groups.

#### Random assignment of subjects

The participants in the study were placed in a rank order based on the Senior Project manager’s understanding of their experience and expertise in underground services. This rank order plus biographical information (e.g., years of experience working on underground services/utilities, years experience using the cable avoidance tool, education level) was used to create stratified pairs of matched subjects. Subjects within each pair were then randomly assigned using a random number generator to either the Control or PBP treatment group. Table 1 shows the biographical data collected on the subjects for the two treatment groups.

**Table 1:** Profile of participants.

**Participants profile (N = 12)**		
The number of years’ experience working on underground services/utilities excavations by study participants		
**Group**	**PBP**	**Control**
0–5 Years	1	1
5–10 Years	3	0
10–20 Years	0	3
20 Years plus	2	2
The number of years’ experience using a cable avoidance tool (or CAT)		
No experience	0	0
0–2 Years	2	1
2–4 Years	1	3
4–6 Years	1	0
6+ Years	2	2
Current level of educational qualification (Highest level of educational achievement noted)		
No Leaving Cert Qualification	4	4
Leaving Cert Qualification	0	0
Craft/vocation qualification	2	0
Third Level Qualification	0	1
Did not respond	0	1

#### Assessment of training outcome

After training, both groups performed the tasks on-site matched assessment periods on three separate occasions. The mean time to perform the scanning and marking was 17 mins (SD = 11). There was no significant difference between the groups (Mann-Whitney U = 97, Z = 1.37, p = 0.18).

After training was completed all subjects completed three real-world trials on the location and excavation of utility services. Each trial was the same distance. The time between trials was determined by the availability of staff to supervise and video record the trial and suitable sites to perform the assessments. It was not possible to impose a predetermined time between each trial but based on previous research we ensured that two trials were completed > 2–3 days apart and < 7 days apart. [15]

Their performance on the tasks were video recorded. Two raters were trained in the use of the assessment checklist until they were able to score a video recorded performance with > 0.8 inter-rater reliability. [6, 16, 17] Post training videos of the procedures were assessed by these two independent subject experts, blinded to individual subjects, training group and procedure order using the procedural checklist list.

#### Real world rollout and implementation of PBP training

In June 2018 we had completed the pilot which involved the 12 subjects in the study. After June a further 38 staff completed the full PBP training program. This included the six workers who participated in the original pilot study but had been randomized to the Control Group. Thus the PBP trainees were made up of, 6 workers from the Control Group in the pilot study, 32 workers new to the program and the 6 PBP trained workers from the pilot study, Total = 44 PBP trained Category 2 workers. In the pilot study all 12 participating workers were RA staff and in the real world PBP rollout implementation 36 workers were RA staff and 8 were subcontractors. Training for all 44 (i.e., 100%) Category 2 workers was mandatory. All workers first completed the e-learning modules used in the pilot study i.e., courses on 1) Job Site Safety Plans 2) Utility Plans and 3) Visual Inspection. They then completed the underground services simulation training (shown in Fig 2a–2c). All workers were required to train until they demonstrated the same quantitatively defined proficiency benchmark as the PBP Group in the pilot study. We assessed the real-world impact of the rollout of the PBP training program in 2018 for all Category 2 workers (n = 44) by comparing the number of utility strikes (i.e., damaging pre-existing utility services) in 2018 in comparison to the previous two years (i.e., 2016–2017) as a function of the number of hours worked (i.e., hours invoiced for Category 2 RA work crews per month).

#### Statistical analysis

A one-way between groups (Current training vs PBP Simulation Training) ANOVA was used to examine mean differences between the outcome measures, i.e., the number of procedural steps required to complete the task, and the number of errors made, by those participating in the simulation-based training programme (Supplementary file 1). In addition the differences between the groups’ performance during the three separate trials were compared using a Fixed Effects Mixed Linear Model in SPSS (version 25). The statistical analysis used was a between groups (i.e., Current Training in comparison to PBP Simulation Training) repeated measures (Trial 1–3) model, with a diagonal error structure.

In order to introduce greater flexibility into the above calculations the remaining results will be done through the use of a statistical model for count data using the real-world data—Poisson regression. This will allow us to control for the number of hours worked, via the use of this variable as an offset measure in the Poisson regression, since this is likely to be a major factor in the number of strikes that occur. The intention will be to examine the effect of an intervention on the number of reported strikes, having controlled for the number of hours worked in each year. The number of strikes made by Category 2RA work crews was recorded on a monthly basis for the years 2016, 2017 and 2018 (Supplementary file 2). In addition, the number of hours contracted to work by Category 2RA work crews was also recorded for each month. The natural log of the hours worked was obtained and this value was used as an offset in the model.

## Results

### Simulation training outcome

**Fig 3:**
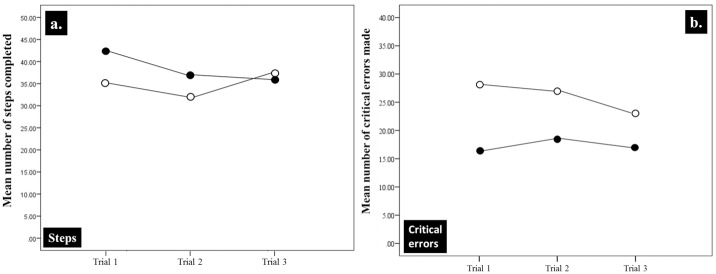
**a**. The mean number of Procedure Steps completed. **b**. Critical Errors made by the two groups across the three assessment trials.

The IRR for all of the video recorded assessments was 0.88 (95% CI: 0.86–0.9). None of the assessments fell below the 0.8 IRR threshold. Fig 3a shows that the PBP group completed more procedure steps than the Control Group in Trials 1 and 2 but approximately the same (i.e., 37 and 38) on Trial 3. Across all three trials the PBP group made fewer critical errors than the Control Group (Fig 3b).

**Fig 4:**
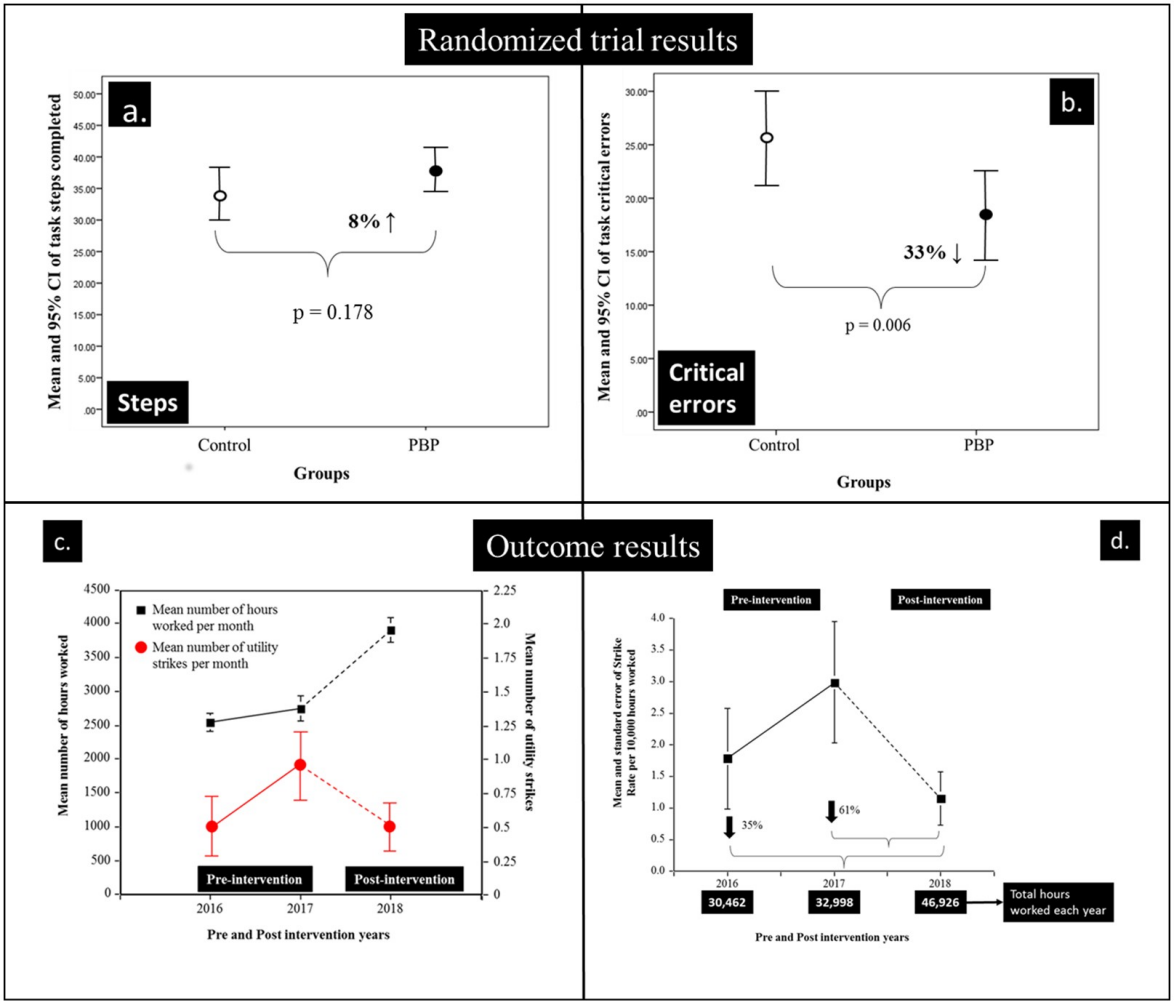
**a**. The mean and 95% confidence intervals (CI) of the number of steps completed the two groups; **b**. The objectively assessed critical errors observed during the video recorded performance trials; **c**. The mean and standard errors of the number of hours worked and utility strikes per month for the years 2016–2018; **d**. The mean and standard errors of the combined utility strike rate per 10,000 hours worked by Category 2 RA work crews in the years 2016–2018.

Fig 4a and 4b summarises the number of procedure steps and critical errors made by the two groups during the three matched assessment periods. A one-way between groups ANOVA for repeated measures was conducted to compare the effect of type of training on the number of procedure steps completed by the two groups. The PBP trained group completed 8% more procedure steps (Fig 4a) than the standard trained control group. Such a difference was not statistically significant (F(1, 30) = 1.713, p = 0.201).

Fig 4b shows that the PBP trained group also made fewer critical errors. On average the Control group made 25.8 critical errors (95% CI: 20.5–31) and the PBP trained group made 17.2 (95% CI: = 13.3–21.1). The difference between the two groups was much larger than for Procedure Steps completed (i.e., 33%) which was found to be statistically significant at the (F(1, 28) = 8.88, p = 0.006). This remained statistically significant in the Mixed Effects Linear Model (t = 3.18, sig = 0.006 (95% CI: 3.24–14.80).

### Assessment of real world PBP training rollout; utility strikes outcomes

The real-world impact of the PBP training program initiated in 2018 was further evaluated by comparing the number of utility strikes (i.e., damaging pre-existing utility services) in comparison to the previous two years 2016–2017. We analysed the number of hours worked (i.e., hours invoiced for Category 2 RA work crews per month).

Fig 4c shows the number of hours worked by Category 2 RA work crews increased across the three years studied. The mean number of hours worked per month in 2016 = 2,539 (95% CI: 2,246–2,831). This increased by 8% in 2017 = 2,750 (95% CI: 2,344–3,155) and increased a further 42% in 2018 = 3,911 (95% CI: 3,500–4,321) which was a 54% increase over the number of hours worked in 2016.

The utility strike rate during the years studied was low. There were no utility strikes in 55.6% of the months studied (i.e., 20 months). There were 11 months with one strike, four months with two strikes and one month with three strikes. There was a total of 23 strikes across the three years studied. The unadjusted mean monthly utility strike was 0.61, the median strike rate = 0 and the mode strike rate = 0. The unadjusted mean monthly strike rate was 0.42 in 2016 which increased to 0.92 in 2017 but dropped back to 0.5 mean strikes per month in 2018.

### Overall standardised incidence rate

The overall average standardised incidence specific rate for hours worked for 2016 (Fig 4d) was 1.64 utility strikes per 10,000 hours worked, (i.e., ∑ months 1 to 12 (strikes/hours worked *10,000)). The equivalent rate in 2017 was 3.33 and in 2018 the rate was 1.28. The standardised incidence rate per 10,000 hours worked was the lowest in 2018 when PBP was implemented in an effort to reduce the number of utility strikes.

### Utility strikes controlling for hours worked: Poisson regression with varying exposure

This alternative approach to calculating the expected incidence rate will again allow us to obtain the standardised incidence ratio, but with a greater level of accuracy since all of the information contained within the data will be used, rather than just the overall average values, as in the above case.

In 2016 the exponentiated value for the standardised incidence ratio is given as 1.28 (Table 2), i.e., the strike rate ratio (SRR) when the result is compared with the reference group, proficiency based progression training in 2018. This result indicates that the number of strikes in 2016 was 28% above those reported in 2018. A similar comparison between the year 2017 and the base year 2018 indicates that the number of strikes was 161% higher (Table 2). In other words, in 2017 the ratio of the number of strikes was 161% higher than in 2018, or to express it differently, there was over one and half times the number of strikes for every 10,000 hours worked in 2017.

**Table 2:** Main results from the Poisson regression statistical analysis of hours worked and strikes made for the years 2016–2018.

		Parameter Estimates								
Parameter	B	Std. Error	95% Wald Confidence Interval		Hypothesis Test			Exp(B)	95% Wald Confidence Interval for Exp(B)	
			Lower	Upper	Wald Chi-Square	df	Sig		Lower	Upper
**(Intercept)**	-8.965	.3467	-9.644	-8.285	668.635	1	.000	.000	6.481E-5	.000
**[Years Data = 2016]**	.250	.5755	-.878	1.378	.188	1	.664	1.284	.416	3.966
**[Years Data = 2017]**	.958	.4363	.103	1.813	4.825	1	.028	2.607	1.109	6.131
**[Years Data = 2018]**	0^a^	.	.	.	.	.	.	1	.	.
**(Scale)**	1^b^									

Dependent Variable: Utility Strikes per month

Model: (Intercept), Year Data (independent variable), offset = LogN (hours worked per month)

^a^. Set to zero because this parameter is redundant.

^b^. Fixed at the displayed value (coefficient relating to the offset).

The results indicate that there was a statistically significant reduction in the number of strikes that occurred when the PBP training group was compared with the incidence of strikes that occurred in 2017. This was statistically significant at the 0.028 level, indicating that the difference in the number of strikes occurring in 2017 had a low probability of having occurred by chance. Hence, it is inferred that the intervention programme was successful in bringing about a reduction in the number of strikes, by >161%. However, we need to be mindful that we are currently dealing with a relatively small sample as indicated by the fairly-wide confidence interval.

It is also evident that though we have a 28% reduction in the number of strikes occurring when the years 2016 and 2018 are compared, the results are not statistically significant at the 0.05 level. The effect is in the ‘right’ direction, but it is evident that a larger sample is required to be able to reliably detect a 28% reduction in strikes, though it must also be kept in mind that the number of hours worked in the year 2016 was the lowest of all of the years. The power to detect the current effect (1.28) is 0.66 with a one-tailed test with an error probability rate of 0.05. The sample would need to be increased by 50% in each condition before a 28% reduction in strike rate could be detected with a power of 0.8.

## Discussion

In this study we found that a PBP training curriculum for the location and excavation of underground utility services resulted in a significant reduction (i.e., 33%) in the objectively assessed critical errors made by utility workers during the prospective and randomised trial. We also found that when the PBP training programme was implemented across all Category 2 RA work crews in 2018 (after completion of the randomized trial) there was a 35% reduction in utility strikes per 10,000 hours worked in comparison to 2016 and a 61% reduction in comparison to 2017. The large reduction in utility strikes observed in 2018 was despite a large and significant increase in the number of hours worked in 2018. The number of hours worked was factored into the statistical analysis of the utility strike reduction but was still found to be statistically significant and unlikely to have occurred by chance.

It should however be noted that the PBP training method was not adopted by all Category 2 RA work crews until after the prospective randomised trial had been completed in June 2018. This means that the reduction observed is probably an underestimation of the impact of PBP training on utility strikes. As a result of the study the PBP skills training and verification methodwill be rolled out to all divisions within the company.

Similar to studies in surgery and cardiology we found that the greatest impact of PBP was on performance errors with a 33% reduction observed in this study [7, 9, 10, 18–22]. These observations have also been verified in well-controlled studies on the transfer of training simulation training [19]. This is an important finding and has fundamental implications for training programmes. It simply means that individuals can score very well on training outcome assessments which only measure whether the task was performed or not. An individual could theoretically score 100% on this type of assessment but this approach to training effectiveness evaluation gives little insight as to how will the task was performed, i.e., the quality of their performance [6]. This is a fundamental problem with training programs per se, even in medicine. For example the Institute of Medicine has concluded that graduate medical education training programs in United States must move away from assessing training effectiveness with process measures such as time training, number of procedures done, number of training courses done etc., to outcome assessments i.e., measuring what the trainee can actually do and how well they can do it [23].

As part of the studies reported here we reliably captured information on the number of utility strikes made by Category 2 RA work crews across the three years. We did not however have reliable information on actual costs incurred by the company as a consequence of the utility strikes. The company were primarily concerned about the safety of its employees and accepted the cost incurred as a consequence of the utility strikes as simply a matter of course. Founded on the work of Makana *et al* (2016) we have estimated that based on the 2016 strike rate (a reduction of ~2 strikes made in 2018) the company saved £116,000–£435,000; if the computations were based on the 2017 strike rate (a reduction of ~10 strikes made in 2018) they saved £580,000 –£2,175,000.

PBP simulation training is effective because of the systematic and conservative approach to learning skills. The skills to be trained are derived from and operationally defined by individuals who are experienced and good at performing the task to be characterised and learned. These performance metrics are then stress tested and validated. Once validated they inform the construction of education and training curricula including simulation platforms. Training outcomes are based on the objectively assessed performance of individuals who are actually good at performing the task rather than benchmarks being estimated [5, 6, 11]. The simulations serve as vehicles for training and honing skilled performance and metrics are used to give formative feedback to trainees during training. This means that they learn what to do and what not to do in a timely fashion, which ensures the training is more efficient and effective. Furthermore, the trainee knows that training is not complete until they have demonstrated the quantitatively defined proficiency benchmark. In our experience this configuration leads to well thought out training programs that are objective, transparent and fair. Furthermore, trainees have a very clear understanding of what is required of them for the training to be completed successfully [6, 8].

In general, it has been found that a PBP approach to training produces improvements in performance of >40% in comparison to current ‘gold standard’ approaches to training [7, 8, 10, 20, 22]. Whilst these benefits have been demonstrated multiple times in surgery, cardiology and anaesthesia this is the first prospective, randomised and blinded study outside medicine to demonstrate that it is equally effective at impacting on safer performance in the utility sector. This study is also the first to demonstrate the real-world impact of a PBP simulation training curriculum on safety and economically impactful events such as utility strikes. The evidence from the study reported here would indicate that the impact is substantial both in terms of the number of strikes avoided by those trained using the PBP simulation training curriculum and the savings made as a result of preventing the strikes £116,000–£2,175,000 based on the more conservative strike cost estimates by Makana et. al. [2].

The costs of implementing a proficiency based progression training programme are however not insubstantial. The procedure characterisation, metric development, operational definition validation and refinement alone cost ~ £0.3m. Additionally, there are the costs of developing and deploying the e-learning platform and curriculum delivered on it as well as the simulation training facility. These are however investments which will significantly add to the scalability of implementing this approach to training across an organization. The greatest expenditure however is in the costs associated with the execution and completion of the prospective randomised and blinded study assessing the effectiveness of this approach to training. The cost of these trials are orders of magnitude greater than all the other costs combined. For profound innovations such as the implementation of PBP simulation training in the utility sector these trials are pivotal. They offer scientifically robust, independent and verifiable evidence from well controlled scientific studies that underpin the acceptance or rejection of this approach. Once completed however, they offer a very robust model and foundation for the development and implementation of other training programs utilising the same scientific approach to the development of simulation-based training and performance benchmarks. The evidence now is very clear that a PBP training method produces skill sets that are significantly better than alternative approaches to training (i.e., >40% better).

### Study limitations

Although we investigated >106,000 work hours this study is still relatively small-scale and focused in one country. The impact of PBP training was assessed in approximately 12 months of follow-up. Even within these constraints the effect size was considerable on detailed performance error assessment in the controlled trial and on utility strikes in the follow-up study. Another limitation was that the research was conducted within one utility organization. This may however have been a strength of the study as the leadership within the organization were very committed to finding a better and safer way to ensure that their staff were as well prepared as possible to identify and avoid potential utility hazards. The aim now is to roll out a PBP approach to training and skill verification across all divisions within the company.

## Conclusions

In a prospective, randomized and blinded study, PBP trained utility workers made significantly fewer (i.e., 33%) objectively assessed task errors in comparison to standard trained utility workers studied. When all of the Category 2 RA workers were trained, it was found to proportionally significantly reduce utility strikes (i.e., by 61%) in comparison to previous years. It was estimated that the cost savings associated with this reduction in utility strikes were between £116,000–£2,175,000 in the 12 month period studied. This is the first well controlled study outside of procedure-based medicine, to use this approach to train skills and safety behaviours. The magnitude of the training outcome in the utilities sector appears to be the same as it is in procedure-based medicine.

## Supporting information

S1 File Prospective, randomized and blinded study data. (PDF)

S2 File Strike rate data compiled Jan 2019. (PDF)

S1 Checklist STROBE statement—Checklist of items that should be included in reports of observational studies. (DOCX)
