# Supplementary material for: Proficiency based progression simulation training significantly reduces utility strikes; A prospective, randomized and blinded study
Source: PLoS One. 2020 May 12;15(5):e0231979. doi: 10.1371/journal.pone.0231979 (PMC7217447; doi:10.1371/journal.pone.0231979)
Supplement: S1 File — (PDF) [file pone.0231979.s001.pdf]

Subject No.	Trial No.	Group	Steps	Critical Errors	Inter rater reliability
1	Trial 1	Control	47	22	0.87
2	Trial 1	Control	32	27	0.87
3	Trial 1	Control	28	25	0.87
4	Trial 1	Control	31	31	0.83
5	Trial 1	Control	25	43	0.87
6	Trial 1	Control	49	17	0.8
7	Trial 1	PBP	41	28	0.8
8	Trial 1	PBP	43	13	0.87
9	Trial 1	PBP	48	12	0.91
10	Trial 1	PBP	45	18	0.91
11	Trial 1	PBP	38	17	0.8
12	Trial 1	PBP	36	11	0.95
1	Trial 2	Control	37	21	0.8
2	Trial 2	Control	40	21	0.83
3	Trial 2	Control	30	30	0.95
4	Trial 2	Control	37	18	0.87
5	Trial 2	Control	19	44	0.91
6	Trial 2	Control			
7	Trial 2	PBP	32	30	0.8
8	Trial 2	PBP	26	26	0.91
9	Trial 2	PBP	44	4	0.91
10	Trial 2	PBP	36	16	0.91
11	Trial 2	PBP	48	15	0.95
12	Trial 2	PBP	35	17	0.91
1	Trial 3	Control	35	33	0.89
2	Trial 3	Control	41	20	0.83
3	Trial 3	Control	38	22	0.95
4	Trial 3	Control	55	5	0.91
5	Trial 3	Control	23	33	0.91
6	Trial 3	Control			
7	Trial 3	PBP	35	21	0.91
8	Trial 3	PBP	21	19	0.95
9	Trial 3	PBP	55	5	0.87
10	Trial 3	PBP	40	27	0.83
11	Trial 3	PBP	34	24	0.95
12	Trial 3	PBP	34	7	0.87
