# Supplementary material for: Proficiency based progression simulation training significantly reduces utility strikes; A prospective, randomized and blinded study
Source: PLoS One. 2020 May 12;15(5):e0231979. doi: 10.1371/journal.pone.0231979 (PMC7217447; doi:10.1371/journal.pone.0231979)
Supplement: S2 File — (PDF) [file pone.0231979.s002.pdf]

Year	Month	Hours worked	Strikes
2016	jan	1929	0
2016	feb	2835	1
2016	mar	3076	0
2016	apr	3293	0
2016	may	2271	1
2016	june	2211	1
2016	jul	2987	0
2016	aug	1967	0
2016	sep	2474	0
2016	oct	2239	2
2016	nov	2270	0
2016	Dec	2910	0
2017	jan	2267	0
2017	feb	2186	1
2017	mar	1853	0
2017	apr	1915	0
2017	may	2584	0
2017	june	3055	1
2017	jul	3074	1
2017	aug	2761	3
2017	sep	3356	1
2017	oct	3432	2
2017	nov	3911	2
2017	Dec	2604	0
2018	jan	4082	1
2018	feb	3627	0
2018	mar	3721	1
2018	apr	3671	0
2018	may	4544	0
2018	june	4750	1
2018	jul	4976	2
2018	aug	3837	0
2018	sep	3884	1
2018	oct	3846	0
2018	nov	3473	0
2018	dec	2515	0
