# Supplementary material for: Proficiency based progression simulation training significantly reduces utility strikes; A prospective, randomized and blinded study
Source: PLoS One. 2020 May 12;15(5):e0231979. doi: 10.1371/journal.pone.0231979 (PMC7217447; doi:10.1371/journal.pone.0231979)
Supplement: S1 Checklist — (DOCX) [file pone.0231979.s003.docx]

STROBE Statement—checklist of items that should be included in reports of observational studies

|  | Item No | Recommendation |
| --- | --- | --- |
| **Title and abstract** | 1 | (*a*) Indicate the study’s design with a commonly used term in the title or the abstract |
|  |  | (*b*) Provide in the abstract an informative and balanced summary of what was done and what was found Page 2 |
| Introduction | | |
| Background/rationale | 2 | Explain the scientific background and rationale for the investigation being reported Page 3-4 |
| Objectives | 3 | State specific objectives, including any prespecified hypotheses Page 5 |
| Methods | | |
| Study design | 4 | Present key elements of study design early in the paper Page 5 Figure 1B |
| Setting | 5 | Describe the setting, locations, and relevant dates, including periods of recruitment, exposure, follow-up, and data collection Page 5-6 |
| Participants | 6 | (*a*) *Cohort study*—Give the eligibility criteria, and the sources and methods of selection of participants. Describe methods of follow-up  *Case-control study*—Give the eligibility criteria, and the sources and methods of case ascertainment and control selection. Give the rationale for the choice of cases and controls Page 7, Table 1  *Cross-sectional study*—Give the eligibility criteria, and the sources and methods of selection of participants |
|  |  | (*b*) *Cohort study*—For matched studies, give matching criteria and number of exposed and unexposed  *Case-control study*—For matched studies, give matching criteria and the number of controls per case |
| Variables | 7 | Clearly define all outcomes, exposures, predictors, potential confounders, and effect modifiers. Give diagnostic criteria, if applicable Page 7 |
| Data sources/ measurement | 8* | For each variable of interest, give sources of data and details of methods of assessment (measurement). Describe comparability of assessment methods if there is more than one group Page 7 |
| Bias | 9 | Describe any efforts to address potential sources of bias |
| Study size | 10 | Explain how the study size was arrived at Page 8-9 |
| Quantitative variables | 11 | Explain how quantitative variables were handled in the analyses. If applicable, describe which groupings were chosen and why Page 9, Fig 3A-D |
| Statistical methods | 12 | (*a*) Describe all statistical methods, including those used to control for confounding Page 8 |
|  |  | (*b*) Describe any methods used to examine subgroups and interactions Page 10-11 |
|  |  | (*c*) Explain how missing data were addressed Not applicable |
|  |  | (*d*) *Cohort study*—If applicable, explain how loss to follow-up was addressed  *Case-control study*—If applicable, explain how matching of cases and controls was addressed  *Cross-sectional study*—If applicable, describe analytical methods taking account of sampling strategy |
|  |  | (*e*) Describe any sensitivity analyses |

Continued on next page

| Results | | |
| --- | --- | --- |
| Participants | 13* | (a) Report numbers of individuals at each stage of study—eg numbers potentially eligible, examined for eligibility, confirmed eligible, included in the study, completing follow-up, and analysed P7 |
|  |  | (b) Give reasons for non-participation at each stage P7 |
|  |  | (c) Consider use of a flow diagram Done |
| Descriptive data | 14* | (a) Give characteristics of study participants (eg demographic, clinical, social) and information on exposures and potential confounders |
|  |  | (b) Indicate number of participants with missing data for each variable of interest |
|  |  | (c) *Cohort study*—Summarise follow-up time (eg, average and total amount) |
| Outcome data | 15* | *Cohort study*—Report numbers of outcome events or summary measures over time |
|  |  | *Case-control study—*Report numbers in each exposure category, or summary measures of exposure Figure 1B |
|  |  | *Cross-sectional study—*Report numbers of outcome events or summary measures |
| Main results | 16 | (*a*) Give unadjusted estimates and, if applicable, confounder-adjusted estimates and their precision (eg, 95% confidence interval). Make clear which confounders were adjusted for and why they were included P10-12 |
|  |  | (*b*) Report category boundaries when continuous variables were categorized Not applicable |
|  |  | (*c*) If relevant, consider translating estimates of relative risk into absolute risk for a meaningful time period |
| Other analyses | 17 | Report other analyses done—eg analyses of subgroups and interactions, and sensitivity analyses P10-12 |
| Discussion | | |
| Key results | 18 | Summarise key results with reference to study objectives P12 |
| Limitations | 19 | Discuss limitations of the study, taking into account sources of potential bias or imprecision. Discuss both direction and magnitude of any potential bias P15 |
| Interpretation | 20 | Give a cautious overall interpretation of results considering objectives, limitations, multiplicity of analyses, results from similar studies, and other relevant evidence P15-16 |
| Generalisability | 21 | Discuss the generalisability (external validity) of the study results P16 |
| Other information | | |
| Funding | 22 | Give the source of funding and the role of the funders for the present study and, if applicable, for the original study on which the present article is based  Martin Hart (MH), David Cleary, Craig Hamilton and Kevin McGlinchey were employees of Reach Active at the time of the study. Patrick Kiely was an employee of Flux Learning Ltd. Neither Reach Active and Flux Learning Ltd. did not play a role in the study design, data collection and analysis, decision to publish, or preparation of the manuscript and only provided financial support in the form of authors' salaries and/or research materials. The study was completed as part of MH’s M.Sc. in Technology Enhanced Learning for Health, at University College Cork, Ireland. The co-authors gave information that facilitated and the study design and data collection (DC, CH, KMcG), built the e-learning Package (PK), expressed their personal opinion on the meaning of the observed results (DC, CH, KMcG, PK), read and gave feedback on the manuscript draft and agreed the final version. |

*Give information separately for cases and controls in case-control studies and, if applicable, for exposed and unexposed groups in cohort and cross-sectional studies.

**Note:** An Explanation and Elaboration article discusses each checklist item and gives methodological background and published examples of transparent reporting. The STROBE checklist is best used in conjunction with this article (freely available on the Web sites of PLoS Medicine at http://www.plosmedicine.org/, Annals of Internal Medicine at http://www.annals.org/, and Epidemiology at http://www.epidem.com/). Information on the STROBE Initiative is available at www.strobe-statement.org.
